# Stakeholder engagement to inform evidence-based treatment implementation for children’s mental health: a scoping review

**DOI:** 10.1186/s43058-022-00327-w

**Published:** 2022-07-29

**Authors:** Noah S. Triplett, Grace S. Woodard, Clara Johnson, Julie K. Nguyen, Rashed AlRasheed, Frank Song, Sophia Stoddard, Jules Cesar Mugisha, Kristen Sievert, Shannon Dorsey

**Affiliations:** 1 Department of Psychology, University of Washington, Guthrie Hall 119A, Box 351525, Seattle, WA 98195 USA; 2 Department of Psychology, University of Miami, P.O. Box 248185, Coral Gables, FL 33124 USA

**Keywords:** Children’s mental health, Evidence-based treatment, Stakeholder engagement

## Abstract

**Background:**

There is a pervasive mental health treatment gap for children across the globe. Engaging stakeholders in child mental health evidence-based treatment (EBT) implementation projects may increase the likelihood of successful EBT implementation, thereby better addressing the treatment gap. However, little is known about the extent of stakeholder engagement to inform the implementation of child mental health EBTs.

**Methods:**

We conducted a scoping review to characterize stakeholder engagement in child mental health EBT implementation projects, including *what* stakeholders are engaged, *how* they are engaged, *when* they are engaged, *where* they are engaged (i.e., location of projects), *why* they are engaged, and the reported *impacts* of stakeholder engagement. We searched seven databases: MEDLINE, PsycInfo, Embase, ERIC, CINAHL Complete, Scopus, and Web of Science Core Collection. To be included, studies had to report on some form of stakeholder engagement that was undertaken to inform or explain the implementation of a child mental health EBT. We performed data extraction and synthesis to describe key study and stakeholder characteristics, stakeholder engagement methods and rationales, reported impacts of stakeholder engagement, and quality of reporting on stakeholder engagement.

**Results:**

In total, 122 manuscripts met our inclusion criteria, from which we identified a total of 103 unique child mental health EBT implementation projects. Projects spanned 22 countries, which included low-, lower-middle, upper-middle, and high-income countries. The largest number of projects was in the USA and conducted in public mental health settings. Most projects engaged EBT providers during the active implementation phase and with limited depth, often gathering information from stakeholders without sharing decision-making power in implementation efforts. Across projects, impacts of stakeholder engagement spanned all of Proctor and colleague’s implementation outcomes.

**Conclusions:**

Given that stakeholder engagement is often shallow and with limited shared decision-making, additional effort should be made to increase engagement to preempt challenges to EBT implementation and ensure implementation success. Such efforts may ensure the just distribution of power in EBT implementation efforts.

**Trial registration:**

All procedures were pre-registered on the Open Science Framework prior to conducting the literature search (DOI 10.17605/OSF.IO/GR9AP).

**Supplementary Information:**

The online version contains supplementary material available at 10.1186/s43058-022-00327-w.

Contributions to the literature

- This is the first scoping review to characterize stakeholder engagement in child mental health EBT implementation projects.
- Providers are most commonly engaged in EBT implementation projects, but there are distinct gaps in the engagement of policymakers, payers, and clients.
- Stakeholder engagement has typically occurred following active implementation to explain determinants of implementation. There is a need to increase depth of engagement with all types of stakeholders by engaging with them earlier in the implementation process and granting them greater decision-making power.
- Across projects, impacts of stakeholder engagement spanned all implementation outcomes. Stakeholder engagement is a potentially powerful strategy to preempt implementation challenges and facilitate initial implementation success.

## Background

Despite the high prevalence of youth mental health disorders and their associated negative impacts, there is a large mental health treatment gap around the globe [1, 2], which is even larger in low- and middle-income countries (LMIC [3, 4];. When youth access mental health care, they often receive minimal evidence-based care, despite the development of numerous evidence-based treatments (EBTs [5–7];). Efforts to support EBT implementation have seen mixed success [8, 9]. The literature often categorizes countries by income due to the large mental health treatment gap in LMIC [3, 4]. In both high-income countries (HIC) and LMIC, slow EBT uptake and implementation have been attributed to implementation challenges that arise when delivering EBTs in complex, real-world settings, such as challenges with funding or lack of organization and provider buy-in [10, 11].

Implementation frameworks emphasize that challenges to EBT implementation may occur with multiple stakeholders across phases of the EBT implementation process [12, 13]. They highlight the importance of stakeholder engagement to preempt these challenges and ensure the relevance and “fit” of an EBT in a given context (e.g., [8, 10]). As defined by the Center for Disease Control and Prevention, stakeholder engagement is “the process of working collaboratively with and through groups of people affiliated by geographic proximity, special interest, or similar situations to address issues affecting the well-being of those people” ([14] p9). Stakeholders themselves can be broadly defined and include clients receiving an EBT, caregivers, community members, providers, agency administrators, payers, and policymakers.

**Fig. 1:**
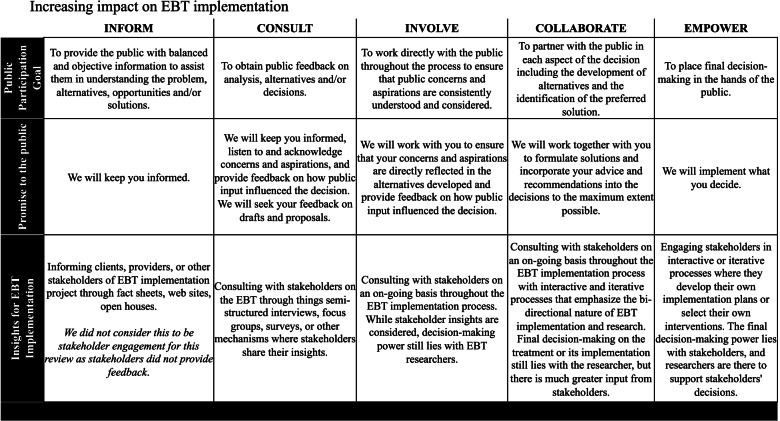
Stakeholder engagement spectrum; adapted from the International Association for Public Participation’s Spectrum of Public Participation

Stakeholders may be engaged in a variety of ways during the EBT implementation process with differing levels of involvement and power. The varying methods of engagement and power shared may have implications for successful EBT implementation. The International Association for Public Participation (IAPP) characterizes stakeholders’ varying levels of engagement and power along a continuum from *inform* (i.e., researchers inform stakeholders of activities) to *empower* (i.e., stakeholders empowered with final decision-making power). This framework is presented in Fig. 1 [15], along with examples of application to child mental health EBT implementation.

On the more collaboration and empowerment-focused end of engagement, research suggests that deeply involving stakeholders in mental health treatment research may increase the impact of research projects [16]. In the USA, Community-Based Participatory Research (CBPR) has become a popular stakeholder-engaged research method that aims to empower community stakeholders as co-investigators at each step of the research process. These methods have facilitated the initial implementation and sustained use of evidence-based health treatments in the USA [17–19]. Though formal CBPR methods are less common in global settings, studies in LMIC have demonstrated that engaging stakeholders can increase the relevance and “fit” of HIC-developed EBTs (e.g., [20]), stakeholder engagement in these contexts has typically involved adapting EBTs for cultural acceptability (e.g., [20, 21]). Given their lived experience, stakeholders often have critical context- and cultural-specific knowledge that historically has not been represented on the research team. This knowledge is essential to adapting EBT content and implementation to ensure successful implementation [20, 21]; yet, little is known about the extent of stakeholder engagement in child mental health EBT implementation projects globally.

Other reviews have characterized the use of formal “research-community partnerships” in evidence-based practice implementation projects [22]; however, more research is needed to characterize the engagement efforts broadly. While some EBT implementation projects may have defined research-community partnerships (e.g., formal agreements about which roles stakeholders play in the research process), others may engage stakeholders in less-defined ways, such as focus groups, interviews, or brainstorming meetings. For youth-focused mental health interventions, in particular, stakeholder engagement is necessitated by the need for buy-in among their caregivers and community members [11]. Access to EBTs for youth often depends on the actions of adult stakeholders, in particular, their caregivers and community members working within service settings in which children are reached [11]. As such, there remains a need to characterize *what*, *how*, *when*, *where*, and *why* stakeholders are engaged, and the reported *impacts* of engaging stakeholders. This scoping review aimed to broadly characterize stakeholder engagement methods used during child mental health EBT implementation projects and recommend future directions. We sought to examine key variables relevant to stakeholder engagement, including types of stakeholders engaged, the implementation phase, the settings, and the method through which stakeholders are typically engaged. Due to the burgeoning stages of this literature, the current review took a more conservative approach and focuses only on peer-reviewed literature, which means that efforts to engage stakeholders that were not published in peer-reviewed journals are not captured.

## Methods

**Fig. 2:**
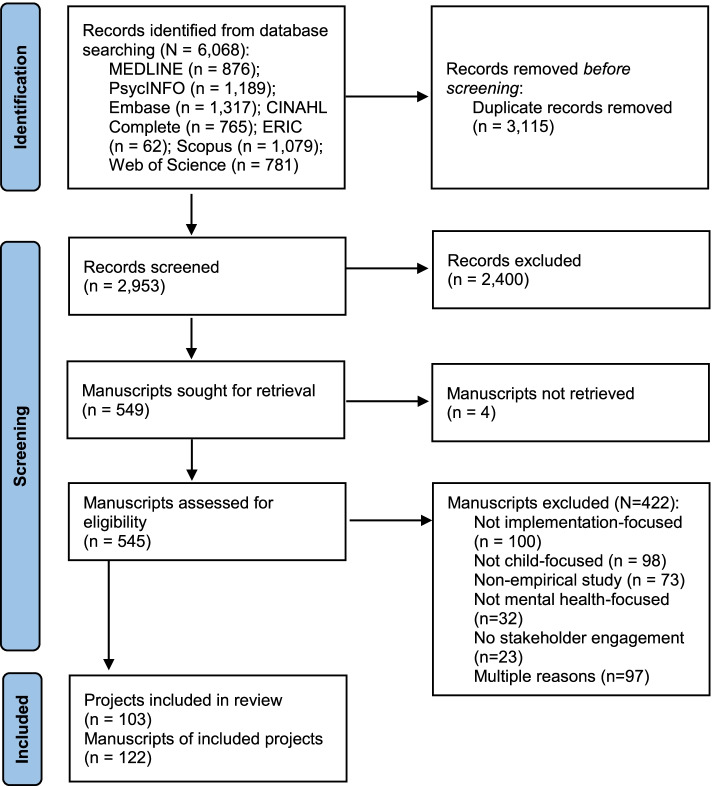
PRISMA flow diagram

Given the heterogeneous nature of stakeholder engagement efforts, as well as the nascent state of the literature that characterizes these efforts, we took a less restrictive approach than a traditional systematic review and conducted a *scoping* review of stakeholder engagement practices [23]. Scoping reviews are indicated over systematic reviews when the existing literature is more varied or emerging [24]. We follow the preferred reporting items for systematic reviews and meta-analyses extension for scoping reviews (PRISMA-ScR) and all procedures were pre-registered on the Open Science Framework [25]. The PRIMSA-ScR flow diagram is presented in Fig. 2, and the checklist is included in Additional file 1.

### Search strategy

We developed a comprehensive search strategy in consultation with a research librarian. To ensure completeness, we reviewed the title, abstract, and keywords from pre-selected articles to generate a list of search terms. We also reviewed search strategies from similar reviews in different disciplines. Search terms were categorized into the following groups: (1) stakeholders, (2) mental health therapy, (3) implementation, and (4) evidence-based practice. All searches were limited to English-language journal articles (not conference abstracts, book chapters, or dissertations). The final search strategy is included in Additional file 2. The first author completed the final search in October 2020. We searched the following seven databases: Medline, PsycInfo, Embase, ERIC, CINAHL Complete, Scopus, and Web of Science Core Collection. We examined the reference lists of studies identified using the search terms above to identify additional articles and check the completeness of our search. The team periodically (though non-systematically) identified and screened additional articles that potentially fit inclusion criteria but were published following the official search date.

### Study inclusion and exclusion criteria

To be selected for review, a study had to describe some form of stakeholder engagement undertaken for informing the implementation of a child mental health-focused EBT. As such, we excluded studies that did not report on implementation processes or outcomes (i.e., we excluded efficacy trials). We excluded broad “evidence-based practice” implementation projects that did not aim to implement specific treatments and EBTs that addressed specific behaviors that may not occur within the context of diagnosable mental health conditions (e.g., problematic sexual behavior). In line with our goals to characterize the practice of stakeholder engagement, we used a broad definition of engagement. It was defined as any attempt to gather perspectives on an intervention or its implementation from people who may interface with the intervention. Given we sought to characterize attempts to gather perspectives from stakeholders, we excluded studies that *informed* stakeholders of EBT implementation projects with no attempt to gather information or hear their perspectives (i.e., the lowest level of engagement on the IAPP framework). Review articles without original data, non-empirical studies, and study protocols were excluded from our review. No other inclusion or exclusion criteria were imposed.

### Study selection

All identified records were imported into a systematic review management tool (Covidence [26]) for abstract and full-text screening as well as extraction. Six reviewers independently assessed study titles and abstracts to determine if they met inclusion criteria. Each study was screened by two reviewers and studies were included for full-text review if both reviewers agreed on inclusion. All reviewers met for twice-monthly consensus meetings to refine inclusion criteria and discuss pre-selected discrepancies. Discrepancies were selected by the first author if they presented opportunities for inclusion criteria refinement and/or reviewer training. Approximately 20% of all discrepancies were resolved in group consensus meetings. Discrepant assessments that were not resolved in consensus meetings were resolved by a third reviewer, who was an experienced coder and masked to the votes of the other two reviewers. After title and abstract screening were completed, eight reviewers independently assessed the full text of selected articles to determine if they met inclusion criteria. Each article was screened by two reviewers, and discrepancies in full-text review were resolved through consensus discussion. When necessary, a third reviewer was consulted to reach consensus.

### Data extraction

A draft extraction table was developed prior to review registration and piloted with a small sample of articles. The extraction table aimed to capture all study characteristics and key elements relevant to stakeholder engagement. Pairs of reviewers extracted data from each study and discussed discrepancies to consensus. Each pair consisted of at least one graduate student with experience conducting systematic reviews. When necessary, a third coder was consulted. The final list of abstracted data is presented in Table 1. We drew from established frameworks to inform data extraction. The items capture study characteristics (i.e., EBT, country, setting), stakeholders engaged, engagement methods (including depth of engagement), implementation phase of engagement, rationale for engagement, any reported effects of engagement on implementation outcomes, and quality of reporting.

**Table 1:** Data extraction

Information extracted	Description
Author	List of authors
Year	Publication year
Title	Study title
Project group	Larger project from which manuscript data originated (if applicable)
EBT	EBT being implemented
Client age	Age of clients treated by the EBT
Country	Country where the study was conducted
World Bank Country Income Classification	World Bank Classifications of Gross National Income per Capita of the Country where the study was conducted
Setting	Physical location where the study was conducted (e.g., mental health agency)
Stakeholders engaged	Number and type of stakeholders engaged
Stakeholder engagement methods	Description of methods used to engage stakeholders
Stakeholder engagement methods category	Classification of methods used to engage stakeholders (qualitative, quantitative, mixed-methods)
Stakeholder engagement rationale	Description of rationale provided for why projects engaged stakeholders
Depth of engagement	Depth of stakeholder engagement, as classified by the International Association for Public Participation’s Spectrum of Public Participation
Phase of implementation	Stage of the EBP implementation (exploration, preparation, implementation, sustainment, multiple, no active implementation, or not reported)
Stakeholder engagement reporting	Presence or absence of Proctor and colleagues’ (2013) specifications for specifying and reporting on implementation strategies
Benefits on implementation outcomes	Reported benefits of stakeholder engagement on Proctor and colleagues’ (2011) implementation outcomes.

*EBT* evidence-based treatment, *EPIS* exploration, preparation, implementation, sustainment framework

Stakeholders were categorized into seven categories: patients/clients, providers, payers, policymakers, community members, caregivers, and researchers. Engagement methods were categorized as follows: quantitative (i.e., surveys), qualitative (e.g., semi-structured interviews or focus groups), mixed-methods, or other (e.g., collaborative workshops without formal qualitative data collection or analysis; advisory boards). We also extracted “rationales” for engaging stakeholders, defined as any mention of why researchers decided to engage with stakeholders at any point in the research process. We adapted the IAPP spectrum of public engagement ( [12]; Fig. 1) to characterize depth of stakeholder engagement in selected studies.

Reviewers also selected the corresponding Exploration, Preparation, Implementation, Sustainment (EPIS; 8) phase(s) in which stakeholders were engaged. We considered stakeholder engagement as a strategy intended to facilitate the implementation of an EBT, and as such assessed the quality of reporting on stakeholder engagement with guidance from Proctor and colleagues’ [27] recommendations on specifying and reporting implementation strategies. We coded the presence or absence of the following details: actor, action, target, temporality, dose, implementation outcome(s) affected, and justification (definitions in Table 2). Each factor in this checklist was coded as present if any aspect of the factor was mentioned, regardless of quality or level of detail. We considered “implementation outcomes(s) affected” as present when studies appropriately identified an implementation outcome, even if they did not measure that outcome or isolate the effect of stakeholder engagement on that outcome. We coded “justification” as present when studies provided pragmatic justifications for their choice of implementation strategy, even if additional empirical or theoretical justifications were not provided. Finally, we extracted the benefits of stakeholder engagement on implementation outcomes, which were defined with Proctor and colleagues’ taxonomy [13].

**Table 2:** Proctor and Colleague’s Outcomes for Implementation Research (2011) and Recommendations for Specifying and Reporting (2013)

Implementation Outcome	Definition
Acceptability	“The perception among implementation stakeholders that a given treatment, service, practice, or innovation is agreeable, palatable, or satisfactory” (Proctor et al., 2011 [13], p. 67)
Adoption	“The intention, initial decision, or action to try or employ an innovation or evidence-based practice” (Proctor et al., 2011 [13], p. 69)
Appropriateness	“The perceived fit, relevance, or compatibility of the innovation or evidence-based practice for a given practice setting, provider, or consumer; and/or perceived fit of the innovation to address a particular issue or problem” (Proctor et al., 2011 [13], p. 69)
Costs	“The cost impact of an implementation effort” (Proctor et al., 2011 [13], p. 69)
Feasibility	“The degree to which an intervention was implemented as it was prescribed in the original protocol or as it was intended by the program developers” (Proctor et al., 2011 [13], p. 69)
Fidelity	“The extent to which a new treatment, or an innovation, can be successfully used or carried out within a given agency or setting” (Proctor et al., 2011 [13], p. 69)
Penetration	“The integration of a practice within a service setting and its subsystems” (Proctor et al., 2011 [13], p. 70)
Sustainability	“The integration of a practice within a service setting and its subsystems” (Proctor et al., 2011 [13], p. 70)
Reporting Specification	Requirements
Actor	“Identify who enacts the strategy (e.g., administrators, payers, providers, patients/consumers, advocates, etc.)” (Proctor et al., 2013 [27], p. 4).
Action	“Use active verb statements to specify the specific actions, steps, or processes that need to be enacted” (Proctor et al., 2013 [27], p. 4).
Action Target	“Specify targets according to conceptual models of implementation” (Proctor et al., 2013 [27], p. 4).
Temporality	“Specify when the strategy is used” (Proctor et al., 2013 [27], p. 4).
Dose	“Specify dosage of implementation strategy” (Proctor et al., 2013 [27], p. 4).
Implementation Outcome Affected	“Identify and measure the implementation outcome(s) likely to be affected by each strategy” (Proctor et al., 2013 [27], p. 4).
Justification	“Provide empirical, theoretical, or pragmatic justification for the choice of implementation strategies” (Proctor et al., 2013 [27], p. 4).

### Data synthesis

Synthesis involved quantitative analysis (e.g., descriptive statistics) of study characteristics and qualitative analysis of stakeholder engagement rationales. When appropriate, we calculated frequencies and percentages for categorical variables (e.g., study characteristics, engagement methods) to broadly characterize stakeholder engagement efforts. We used an inductive approach to qualitatively code rationale of stakeholder engagement. The first and second authors independently reviewed all reported rationales for stakeholder engagement and generated themes that emerged from the reported rationales. We present frequencies of themes across reported rationales. Some rationales were coded as multiple themes. Across all data elements, we split results by LMIC and HIC.

## Results

The search yielded a total of 6068 articles. After excluding duplicates, 2953 titles and abstracts were reviewed for inclusion. Among those, 545 articles progressed to full-text review and 122 articles met criteria for inclusion (Fig. 2). Articles were primarily excluded because they did not report on implementation processes or outcomes (*n* = 100), or they were not child-focused (*n* = 98). We identified a total of 103 unique child mental health EBT implementation projects across all 122 included articles. To capture the full range of stakeholder engagement in an implementation project, which may be segmented into multiple manuscripts, data from the same projects were clustered together. Each project is represented only once in all results.

### Descriptive findings

#### Study setting

Study characteristics and descriptive findings are summarized in Table 3. Projects were conducted across 22 countries. Countries varied with regard to their gross national income, as classified by the World Bank Atlas method [28]. Most projects were conducted in high-income countries (*n* = 85; 82.5%), with smaller numbers conducted in upper-middle-income (*n* = 5; 4.9%), lower-middle-income (*n* = 10; 9.7%), and low-income countries (*n* = 3; 2.9%). Across all countries, EBTs were most frequently delivered in community mental health settings (*n* = 44; 42.7%), followed by schools (*n* = 27; 26.2%). There were notable differences in settings between projects conducted in HIC (*n* = 85) and LMIC (*n* = 18). Most projects in HIC were in community mental health settings (*n* = 43; 50.6%), followed by schools (*n* = 21; 24.7%) and child welfare centers (*n* = 14; 16.5%). By contrast, when examining projects that occurred in LMIC (*n* = 18), EBTs were most often delivered in school settings (*n* = 6; 33.3%) or other community or non-profit settings (*n* = 9; 50.0%).

**Table 3:** Study characteristics and descriptive statistics by country income

	Low-to-middle-income countries (***n*** = 18)		High-income countries (***n*** = 85)		Overall (***N***=103)	
**Continent**						
North America	1	5.6%	78	91.8%	79	76.7%
Africa	10	55.6%	—		10	9.7%
Asia	5	27.8%	2	2.4%	7	6.8%
Europe	1	5.6%	3	3.5%	4	3.9%
Australia	—		2	2.4%	2	1.9%
South America	1	5.6%	—		1	1.0%
**Setting**						
Community mental health	1	5.6%	43	50.6%	44	42.7%
Schools	6	33.3%	21	24.7%	27	26.2%
Hospitals	—	—	6	7.1%	6	5.8%
Juvenile justice	—	—	3	3.5%	3	2.9%
Child welfare centers	1	5.6%	14	16.5%	15	14.6%
Substance use	—	—	1	1.2%	1	1.0%
Other	9	50.0%	6	7.1%	15	14.6%
Not reported	2	11.1%	5	5.9%	7	6.8%
**Stakeholders engaged**						
Patients/clients	6	33.3%	16	18.8%	22	21.4%
Providers	16	88.9%	69	81.2%	85	82.5%
Private payers	—	—	1	1.2%	1	1.0%
Policymakers	4	22.2%	14	16.5%	18	17.5%
Community members	9	50.0%	19	22.4%	28	27.2%
Caregivers	9	50.0%	32	37.6%	41	39.8%
Researchers	4	22.2%	20	23.5%	24	23.3%
Agency administrators	4	22.2%	49	57.6%	53	51.5%
**EPIS Phase**						
Exploration	8	44.4%	19	22.4%	27	26.2%
Preparation	6	33.3%	42	49.4%	48	46.6%
Implementation	14	77.8%	65	76.5%	79	76.7%
Sustainment	1	5.6%	11	12.9%	12	11.7%
**Rationale**						
Build partnership	4	22.2%	24	28.2%	28	27.2%
Inform	10	55.6%	29	34.1%	39	37.9%
Explain	8	44.4%	46	54.1%	54	52.4%
Not reported	1	5.6%	7	8.2%	8	7.8%
**Methods**						
Quantitative	1	5.6%	20	23.5%	21	20.4%
Qualitative	7	38.9%	27	31.8%	34	33.0%
Mixed-methods	7	38.9%	29	34.1%	36	35.0%
Other	3	16.7%	21	24.7%	24	23.3%
**Depth of engagement**						
Consult	6	33.3%	37	43.5%	43	41.7%
Involve	4	22.2%	19	22.4%	23	22.3%
Collaborate	5	27.8%	18	21.2%	23	22.3%
Empower	3	16.7%	11	12.9%	14	13.6%
**Impacts by implementation outcome**						
Acceptability	7	38.9%	9	10.6%	16	15.5%
Feasibility	5	27.8%	7	8.2%	12	11.7%
Fidelity	1	5.6%	4	4.7%	5	4.9%
Penetration	—	—	6	7.1%	6	5.8%
Adoption	2	11.1%	4	4.7%	6	5.8%
Appropriateness	—	—	3	3.5%	3	2.9%
Cost	—	—	1	1.2%	1	1.0%
Sustainability	—	—	1	1.2%	1	1.0%
**Reporting on stakeholder engagement**						
Actor	15	83.3%	69	81.2%	84	81.6%
Action	18	100.0%	79	92.9%	97	94.2%
Target	18	100.0%	82	96.5%	100	97.1%
Temporality	16	88.9%	76	89.4%	92	89.3%
Dose	8	44.4%	56	65.9%	64	62.1%
Implementation outcome	13	72.2%	43	50.6%	56	54.4%
Justification	18	100.0%	78	91.8%	96	93.2%

*EPIS* exploration, preparation, implementation, sustainment framework

#### Stakeholders engaged

Across all countries, projects most frequently engaged with providers (*n* = 85; 82.5%), followed by agency administrators or other staff (*n* = 53; 51.5%) and children’s caregivers (*n* = 41; 39.8%). Similar to overall patterns, projects in HIC most frequently engaged with providers (*n* = 69; 81.2%), followed by agency administrators or other staff (*n* = 49; 57.6%) and children’s caregivers (*n* = 32; 37.6%). In contrast, projects in LMIC (*n* = 18) nearly always engaged providers (*n* = 16; 88.9%) and frequently engaged community members (*n* = 9; 50.0%) and caregivers (*n* = 9; 50.0%). Projects in LMIC also more frequently engaged with clients (*n* = 6; 33.3%) than projects in HIC (*n* = 16; 18.8%).

#### EPIS phase of engagement

Across all projects, stakeholders were most frequently engaged during the active implementation EPIS phase (*n* = 79; 76.7%), followed by the preparation (*n* = 48; 46.6%) and exploration phases (*n* = 27; 26.2%). Projects in HIC (*n* = 86) followed a similar pattern to our overall results, with engagement most frequently occurring in the active implementation (*n* = 65; 76.5%) and preparation EPIS phases (*n* = 42; 49.4%). Again, contrasting projects conducted in LMIC (*n* = 18), stakeholders were most often engaged during the active implementation EPIS phase (*n* = 14; 77.8%). However, projects in LMIC more frequently engaged stakeholders in the exploration EPIS phase (*n* = 8; 44.4%) than projects in HIC (*n* = 19; 22.4%).

#### Rationale

Ninety-five projects (92.2% of total) described a rationale for engaging stakeholders. Rationales clustered under three themes: building partnerships; informing subsequent implementation; and explaining implementation processes following implementation. Twenty-eight projects (27.2% of total) reported that a rationale for their engagement with stakeholders was to engage with communities and build partnerships to support EBT implementation. These efforts were often in the context of implementation planning and EBT adaptation; however, some studies discussed desires to build partnerships generally and not within the context of implementation or adaptation. Thirty-nine projects (37.9%) reported that a rationale for engaging stakeholders was to inform the subsequent implementation of EBTs, such as through implementation planning or EBT adaptation. Finally, 54 projects (52.4%) reported engaging stakeholders to explain barriers and facilitators that were encountered during the implementation of EBTs. Rationales were similar across projects conducted in HIC and LMIC; however, projects conducted in LMIC more frequently reported a rationale of engaging stakeholders to inform subsequent implementation (*n* = 10; 55.6%) than projects conducted in HIC (*n* = 29; 34.1%).

#### Methods

Stakeholders were engaged in a variety of different ways, including through mixed-methods (*n* = 36; 35.0%), qualitative interviews or focus groups (*n* = 34; 33.0%), and questionnaires (*n* = 21; 20.4%). Many projects also engaged stakeholders without formal data collection (*n* = 24; 23.3%), through processes such as community advisory boards, collaborative planning meetings, and formal CBPR methods. Notably, mixed-methods (*n* = 7; 38.9%) and qualitative interviews or focus groups (*n* = 7; 41.2%) were common in projects conducted in LMIC, but exclusive use of quantitative methods (i.e., questionnaires and surveys) was not (*n* = 1; 5.6%). In contrast, nearly one quarter (*n* = 20; 23.5%) of projects in HIC relied exclusively on quantitative methods to engage stakeholders.

#### Depth of engagement

Overall, projects most often *consulted* with stakeholders (*n* = 43; 41.7%), meaning they obtained feedback from stakeholders through interviews or surveys on one occasion with no follow-up or shared decision-making. Fewer numbers of projects *involved* (*n* = 23; 22.3%) or *collaborated* (*n* = 23; 22.3%) with stakeholders. *Involvement* required multiple solicitations of feedback through interviews, focus groups, or surveys, but again, granted limited decision-making power to stakeholders. *Collaboration* indicated a greater amount of power was given to stakeholders, such as by allowing stakeholders to participate in shared decision-making meetings to adapt EBTs or implementation processes. The fewest number of studies *empowered* stakeholders (*n* = 14; 13.6%), meaning they placed the final decision-making power in the hands of the stakeholder through processes like iterative implementation plan development or EBT selection. Notably, projects conducted in LMIC less frequently consulted with stakeholders (33.3% v. 43.5%) and more frequently empowered stakeholders (16.7% v. 12.9%) than projects in HIC.

#### Impacts of stakeholder engagement

Only 32 studies (31.1%) discussed impacts of stakeholder engagement on implementation outcomes. All discussed impacts were in terms of benefits on EBT implementation. Studies typically reported on implementation outcomes following stakeholder engagement methods and highlighted how engagement efforts might have impacted implementation outcomes. For example, studies frequently engaged stakeholders to adapt EBT content and then conducted assessments of the acceptability, feasibility, or other outcomes of the adapted EBT. Projects conducted in LMIC more frequently reported impacts of stakeholder engagement (*n* = 8; 44.4%) than projects conducted in HIC (*n* = 24; 28.2%). Of the 32 studies that discussed impacts of stakeholder engagement, the most reported impacts were on EBT acceptability (*n* = 16; 50.0%) and feasibility (*n* = 12; 37.5%).

#### Reporting on stakeholder engagement

The reporting of stakeholder engagement varied significantly across studies in our sample, with few differences between projects conducted in LMIC and HIC. Overall, *actors* (i.e., the individuals conducting the engagement efforts) were reported in most projects (*n* = 84; 81.6%), as were the *actions* taken to engage stakeholders (*n* = 97; 94.2%). However, the level of detail provided on actions to engage stakeholders varied between studies, with some describing their procedures in-depth and others briefly summarizing engagement strategies (e.g., “shared decision-making meetings”). There was an apparent difference in reporting dose between projects in HIC (*n* = 56; 65.9%) and those in LMIC (*n* = 8; 44.4%). Whereas temporality could be inferred from the stage of the project in which engagement occurred (e.g., post-implementation), studies frequently omitted details about how long focus groups, semi-structured interviews, or other engagement methods lasted. *Justification* (i.e., justifying why projects chose to engage stakeholders) was often inferred from studies’ rationales for engagement and frequently reported (*n* = 96; 93.2%). However, studies least frequently reported on the *implementation outcomes affected* or likely to be affected by stakeholder engagement (*n* = 56; 54.4%). Interestingly, though, studies in LMIC more frequently reported implementation outcomes affected (*n* = 13; 72.2%) than projects conducted in HIC (*n* = 43; 50.6%).*Examining connections between stakeholders engaged, depth, and methods of engagement.*

Table 4 presents the breakdown of depth of engagement and EPIS phase in which each type of stakeholder was engaged. Notably, this is presented at the project level and may not capture within-project variation in engagement (i.e., projects may have engaged with one stakeholder group during one EPIS phase and another group at a different EPIS phase or projects may have granted some stakeholders more power than others in the engagement process). Client and provider engagement does appear to be slightly skewed toward lower levels of engagement (i.e., projects most often only *consult* with clients and providers). This pattern does not hold for other stakeholders. In terms of EPIS phase of engagement, it appears policymakers and community members are engaged earlier in the implementation process than most other stakeholders, including clients and providers.

**Table 4:** Stakeholders engaged, depth of engagement, and EPIS phase of engagement

	**Patients/clients (** ***n*** **=22)**		**Providers (** ***n*** **=85)**		**Private payers (** ***n*** **=1)**		**Policymakers (** ***n*** **=18)**		**Community members (** ***n*** **=28)**		**Caregivers (** ***n*** **=41)**		**Researchers (** ***n*** **=24)**		**Agency admin (** ***n*** **=53)**	
**Depth of engagement**																
Consult	9	40.9%	33	38.8%	1	100.0%	5	27.8%	6	21.4%	13	31.7%	5	20.8%	14	26.4%
Involve	6	27.3%	19	22.4%	—	—	4	22.2%	7	25.0%	13	31.7%	7	29.2%	12	22.6%
Collaborate	5	22.7%	21	24.7%	—	—	8	44.4%	12	42.9%	12	29.3%	9	37.5%	15	28.3%
Empower	2	9.1%	12	14.1%	—	—	1	5.6%	3	10.7%	3	7.3%	3	12.5%	12	22.6%
**EPIS phase** ^a^																
Exploration	6	27.3%	23	27.1%	—	—	9	50.0%	15	53.6%	12	29.3%	12	50.0%	16	30.2%
Preparation	8	36.4%	44	51.8%	—	—	11	61.1%	20	71.4%	20	48.8%	17	70.8%	33	62.3%
Implementation	19	86.4%	65	76.5%	1	100.0%	12	66.7%	19	67.9%	32	78.0%	15	62.5%	40	75.5%
Sustainment	3	13.6%	9	10.6%	—	—	2	11.1%	2	7.1%	4	9.8%	3	12.5%	7	13.2%

*EPIS* exploration, preparation, implementation, sustainment framework

^a^Categories are not mutually exclusive, so percentage and totals may exceed 100%

Table 5 presents the breakdown of EPIS phase, rationale, and methods by depth of engagement. Again, this is presented at the project level and may not capture within-project variation in engagement. Greater depth of stakeholder engagement tended to be associated with higher involvement throughout EPIS phases. While engagement during the active implementation phase was common for all levels of depth, projects that *collaborated* with and *empowered* stakeholders more frequently engaged with stakeholders during the exploration, preparation, and sustainment phases. Similar patterns emerged with regard to rationale, wherein projects that aimed to explain barriers and facilitators during implementation less frequently *collaborated* with and *empowered* stakeholders as compared to projects that aimed to build partnerships or inform implementation. Finally, qualitative, mixed-method, and other participatory research approaches tended to be more common in projects that *collaborated* with and *empowered* stakeholders.

**Table 5:** Associations between rationale, methods, and depth of stakeholder engagement

	**Consult (*n*** **= 43)**		**Involve (*n*** **= 23)**		**Collaborate (*n*** **= 23)**		**Empower (*n*** **= 14)**	
**EPIS phase**^a^								
Exploration	3	7.0%	8	34.8%	10	43.5%	6	42.9%
Preparation	7	16.3%	13	56.5%	19	82.6%	9	64.3%
Implementation	32	74.4%	18	78.3%	17	73.9%	12	85.7%
Sustainment	6	14.0%	2	8.7%	0	0.0%	4	28.6%
**Rationale**^a^								
Build Partnership	6	14.0%	9	39.1%	7	30.4%	6	42.9%
Inform	9	20.9%	11	47.8%	13	56.5%	6	42.9%
Explain	32	74.4%	8	34.8%	6	26.1%	8	57.1%
Not reported	2	4.7%	3	13.0%	3	13.0%	—	—
**Methods**^a^								
Quantitative	12	27.9%	4	17.4%	4	17.4%	1	7.1%
Qualitative	14	32.6%	8	34.8%	10	43.5%	2	14.3%
Mixed-methods	14	32.6%	7	30.4%	6	26.1%	9	64.3%
Other	4	9.3%	6	26.1%	11	47.8%	3	21.4%

*EPIS* exploration, preparation, implementation, sustainment framework

^a^Categories are not mutually exclusive, so percentage and totals may exceed 100%

## Discussion

This scoping review aimed to synthesize how stakeholders have been engaged in child mental health EBT implementation projects. We identified and examined studies from 22 different countries spanning all four World Bank income classifications. Despite the burgeoning literature that suggests deep engagement of various stakeholder groups within each phase of the implementation process is critical, the majority of studies examined in this review do not report such engagement.

Our synthesis suggests that some critical groups of stakeholders—policymakers, private payers, and clients—may be less frequently engaged in the EBT implementation process. Research has documented the myriad of challenges that can arise at the policy and financial levels that impede the successful implementation EBTs [29–31]. There have been growing efforts to engage with policymakers to generate evidence-informed policy that supports EBT implementation and sustained use [32]; however, there is less scholarship on engaging with private payers and other funders. Engaging policymakers and private payers throughout child mental health EBT implementation projects may address the myriad of financial challenges that organizations face when implementing and sustaining EBTs [33]. Relatedly, engaging clients may be another vital tool to improve the implementation of EBTs. In our synthesis, client and provider engagement appeared to be skewed toward lower levels of engagement. Despite challenges associated with engaging youth clients in mental health research [34], youth involvement is crucial to ensure that projects are relevant and responsive to youth needs [35]. A growing body of research has focused on engaging clients in improving or redesigning mental health service delivery with a recent systematic review noting the potential benefits of engaging clients to enhance quality of care [36]. Moving forward, EBT implementation projects should continue to broaden their engagement efforts beyond providers, with a focus on including payers and clients. Such efforts may improve both the success of EBT implementation and the likelihood of sustainment through supporting policy and funding.

Distinct patterns emerged in terms of stakeholders engaged, depth, and methods of engagement. Greater depth of stakeholder engagement tended to be associated with higher involvement throughout EPIS phases. Projects that *collaborated* with and *empowered* stakeholders more frequently engaged with stakeholders during the exploration, preparation, and sustainment phases—meaning stakeholders maintained influence throughout the project. This was reflected in rationale and methods as well, where we saw that *empowering* projects sought to partner with stakeholders and utilized qualitative or mixed-mixed approaches to do so. As researchers and partners continue to implement EBTs, projects should consider and define their rationales for engaging with stakeholders and ensure their methods and level of shared decision-making is reflective of their rationale.

Notably, there were some key differences between projects in LMIC and HIC. Projects in LMIC more frequently engaged with stakeholders earlier in the implementation process, including to select and adapt EBTs and more frequently reported impact of engagement on EBT feasibility and acceptability. Given that most EBTs were developed and have been primarily tested in HIC, this likely reflects the need to adapt EBTs and engage stakeholders for crucial context- and cultural-specific knowledge. Engaging stakeholders in HIC earlier in the EBT implementation process (e.g., when deciding which EBTs to implement or preparing to begin implementation) may similarly provide crucial knowledge that preempts some barriers to EBT implementation and increases the likelihood of success. There is still great value in engaging stakeholders later in the EBT implementation process, such as after active implementation has already begun, to assess and refine implementation. However, a key benefit of stakeholder engagement may be the knowledge and buy-in gained before implementation that can help researchers and implementing partners more successfully initially implement EBTs.

Of the 103 projects, we identified two highly participatory projects that demonstrate the various ways in which stakeholders can be engaged throughout the EBT implementation process. These are exemplars that may guide other projects to engage stakeholders throughout the implementation process. The “An Individualized Mental Health Intervention for Autism Spectrum Disorder (AIM HI)” project represents a years-long partnership that *empowered* stakeholders (caregivers, providers, agency administrators) to first understand barriers to treating autism in community mental health settings [37, 38] then collaboratively develop an EBT protocol and therapist training model [39]. Following development and pilot testing [39], the AIM HI project engaged stakeholders to examine therapist [40, 41] and parent [42, 43] perspectives of the intervention as well as guide systematic adaptation of the intervention for Latinx families [44]. In another project implementing the Cognitive Behavioral Intervention for Trauma in Schools (CBITS [45]), Kataoka and colleagues [46] *empowered* stakeholders through their community-participatory work to address mental health problems of children exposed to violence. Stakeholders (researchers; providers; caregivers; agency administrators; community members) were engaged throughout the implementation process and discussed all aspects of the project, “including the research design, recruitment, intervention components, and adapting existing services to complement the [intervention]” ( [44] p91). To begin, stakeholders and researchers drafted a memorandum of understanding to define their partnership. Stakeholders and researchers then jointly decided on CBITS and collaboratively adapted it. These two projects notably began stakeholder engagement early in the process, which allowed them to adapt to feedback and grant stakeholders more power in the process. These efforts may not only be essential to ensuring initial implementation efforts are more successful but also to ensuring that stakeholders are valued as equal partners in EBT implementation efforts.

The extent and quality of the reporting on stakeholder engagement were variable within our included projects. Overall, the quality of reporting on stakeholder engagement was high—a majority of projects reported on all aspects of Proctor and colleagues [27] rubric. However, there was significant variation by reporting element. Implementation outcomes likely to be affected by stakeholder engagement were among the least reported, with slightly more than half of the projects (54.4%) linking stakeholder engagement efforts to a specific implementation outcome regardless of if they actually measured that implementation outcome. This finding may be explained in part by studies that were published before the publishing of Proctor and colleagues’ guidelines in 2013 (*n* = 21; 20.4%). Further, some projects may not consider stakeholder engagement as a discrete implementation strategy and therefore may not have considered its impacts within the context of defined implementation outcomes. Despite this, conceptualizing stakeholder engagement as an implementation strategy and improving reporting on it, specifically in relation to implementation outcomes likely to be affected, may influence other researchers to engage stakeholders. Ensuring efforts are specified such that stakeholder engagement can be replicated with other projects is essential to increasing engagement of stakeholders within child mental health EBT implementation projects.

Finally, relatively few projects reported impacts of stakeholder engagement. Given the aims of this review (i.e., to characterize stakeholder engagement methods broadly) and our inclusion criteria, studies were not required to isolate the effects of stakeholder engagement on implementation outcomes or explicitly discuss the impacts of stakeholder engagement on implementation outcomes. Reported impacts were most frequent in terms of increased acceptability and feasibility. For example, Rose-Clarke and colleagues [47] engaged stakeholders to inform the implementation of group interpersonal therapy for adolescents with depression in rural Nepal. They reported adaptations to “optimize treatment delivery” and “emphasize developmental and cultural aspects of depression,” such as “integrating therapy into secondary schools for delivery by school nurses and lay community members … ” and “using locally acceptable terms for mental illness … ” ( [46] p12). Projects also discussed the impacts of stakeholder engagement on other implementation outcomes, including adoption and penetration. Donenberg and colleagues’ [48] discussed the impacts of engaging stakeholders during the exploration phase to “develop a genuine community-engaged approach … ” that “laid the foundation for widescale buy-in” to a cognitive behavioral therapy intervention intended to improve mental health symptoms and antiretroviral therapy adherence for Rwandan adolescents living with HIV. Though projects did not frequently discuss the impacts of stakeholder engagement on implementation outcomes, we caution from inferring from this synthesis that stakeholder engagement does not have substantial impact on implementation. The noted lack of discussion of impacts of stakeholder engagement is likely a result of the current scope of stakeholder engagement in EBT implementation projects, in which stakeholders are typically engaged following the initial implementation to *explain* barriers. As stakeholders continue to be involved in EBT implementation efforts, hopefully with greater depth and earlier in the implementation process, further work is needed to outline the full extent of impacts of stakeholder engagement on EBT implementation.

Given the varied findings from this scoping review and the extant literature indicating the importance of stakeholder engagement, we provide suggestions for future researchers. First and foremost, stakeholders must be engaged to some extent in every implementation study. The types of stakeholders and timing of engagement will likely depend on multiple factors including budget, resources, and the development stage of an intervention being implemented. In general, the more types of stakeholders involved, the better-positioned researchers may be to provide practical guidance for future implementation. We also suggest that stakeholders are involved in each aspect of the implementation process and that they are empowered to contribute ideas and decisions. To increase the use, depth, and reporting of engagement overall, we suggest that researchers collaborate with those who are empowering stakeholders throughout the implementation process. Much can be learned from studies like AIM HI and CBITS. In addition, our findings that projects in LMIC more frequently involve stakeholders earlier in implementation process, empower stakeholders, and report engagement impact on implementation outcomes than HIC projects indicate that research in LMIC can guide stakeholder engagement in HIC.

### Limitations of the current study

Our findings should be considered within the context of some limitations. First, we only examined peer-reviewed empirical studies. As such, any study protocols or non-peer-reviewed articles were excluded. We acknowledge that some researchers may choose to outline engagement methods or existing partnerships in study protocols. Additionally, publicly funded EBT implementation projects may report results in gray literature (i.e., reports), which were not captured in our review. Second, we recognize that our ability to review stakeholder engagement is dependent on the extent to which these activities were reported. It is possible that more stakeholder engagement occurred than was reported in our included studies. Improved reporting of stakeholder engagement activities, following recommended standards for specifying and reporting on implementation strategies [27] may help to elucidate the full extent of stakeholder engagement in implementation efforts. Finally, our review may not adequately describe the impacts of stakeholder engagement. Stakeholder engagement was generally one piece of a much larger implementation effort, and implementation outcomes were usually discussed in terms of the full project. As such, aside from specific mentions of program adaptations that were informed by stakeholders to improve acceptability, extraction was not able to discern the specific impact of stakeholder engagement on implementation outcomes.

## Conclusion

While many different types of stakeholders have been engaged in EBT implementation projects, they are typically engaged following the initial implementation to explain implementation barriers and facilitators. Stakeholders were generally not engaged with a degree of depth that would allow them to influence key decisions in the EBT implementation process. Key steps to improve stakeholder engagement efforts and advance our understanding of its impacts include increasing variety of stakeholders engaged to include clients and payers; engaging stakeholders earlier in the implementation process and granting them more decision-making power; comprehensively reporting on stakeholder engagement activities; and exploring and documenting the impacts of stakeholder engagement on EBT implementation outcomes.

## Supplementary Information

**Additional file 1.**

**Additional file 2.**

## Data Availability

All articles included in this systematic review are publicly available. The datasets used and/or analyzed during the current study are available from the corresponding author on reasonable request.

## Abbreviations

AIM HI: An Individualized Mental Health Intervention for Autism Spectrum Disorder
CBPR: Community-Based Participatory Research
CBITS: Cognitive Behavioral Intervention for Trauma in Schools
EBT: Evidence-based treatment
EPIS: Exploration, Preparation, Implementation, Sustainment
HIC: High-income countries
HIV: Human immunodeficiency virus
LMIC: Low- to middle-income countries
PRISMA-ScR: Preferred reporting items for systematic reviews and meta-analyses extension for scoping reviews
