# Supplementary material for: Stakeholder engagement to inform evidence-based treatment implementation for children’s mental health: a scoping review
Source: Implement Sci Commun. 2022 Jul 29;3:82. doi: 10.1186/s43058-022-00327-w (PMC9338493; doi:10.1186/s43058-022-00327-w)
Supplement: Supplementary file 2 — Additional file 2. [file 43058_2022_327_MOESM2_ESM.docx]

**Final Search Strategies**

These strategies are designed to capture articles discussing stakeholders, mental health therapy, implementation, and evidence-based practice. All searches were limited to English-language journal articles (not conference abstracts, book chapters, or dissertations).

Search strategies are provided for 7 databases: Medline (Ebsco), PsycInfo (Ebsco), Embase (Elsevier), ERIC (Ebsco), CINAHL Complete (Ebsco), Scopus, Web of Science Core Collection

**Medline (Ebsco) – 876 references**

MH “Community Participation+” OR MH “Patient Participation+” OR MH “Caregivers” OR TI (Stakeholder OR stakeholders OR “key informant” OR “key informants” OR “engagement framework” OR “engagement method” OR “facilitating engagement” OR “community academic partnership” OR “community advocate” OR “community advocates”) OR AB (Stakeholder OR stakeholders OR “key informant” OR “key informants” OR “engagement framework” OR “engagement method” OR “facilitating engagement” OR “community academic partnership” OR “community advocate” OR “community advocates”) OR TI ((patient OR patients OR consumer OR consumers OR client OR clients OR public OR community OR communities OR customer OR customers OR agency OR agencies OR government* OR payer OR payers OR purchaser* OR insurance OR medicare OR Medicaid OR administrator* OR physician* OR clinician* OR provider* OR layperson* OR laypeople OR lay OR caregiver* OR employer*) N3 (engage* OR engaging OR collaborat* OR participat* OR partner* OR involve* OR input)) OR AB ((patient OR patients OR consumer OR consumers OR client OR clients OR public OR community OR communities OR customer OR customers OR agency OR agencies OR government* OR payer OR payers OR purchaser* OR insurance OR medicare OR Medicaid OR administrator* OR physician* OR clinician* OR provider* OR layperson* OR laypeople OR lay OR caregiver* OR employer*) N3 (engage* OR engaging OR collaborat* OR participat* OR partner* OR involve* OR input))

**AND**

MH “Mental Health Services+” OR MH “Psychotherapy+” OR MH "Psychological Techniques+" OR MH "Psychiatric Somatic Therapies+" OR TI (psychotherapy OR “behavioral therapy” OR “behavior therapy” OR “behaviour therapy” OR “behavioural therapy” OR “mental healthcare” OR “behavioral healthcare” OR “behavioural healthcare” OR (psychologic* OR psychiatric OR psychosocial OR behavioral OR behavioural OR “mental health” OR addiction) N5 (intervention OR treatment OR treatments OR therapy OR therapies OR care OR healthcare OR support OR practice OR practices OR service OR services)) OR AB (psychotherapy OR “behavioral therapy” OR “behavior therapy” OR “behaviour therapy” OR “behavioural therapy” OR “mental healthcare” OR “behavioral healthcare” OR “behavioural healthcare” OR (psychologic* OR psychiatric OR psychosocial OR behavioral OR behavioural OR “mental health” OR addiction) N5 (intervention OR treatment OR treatments OR therapy OR therapies OR care OR healthcare OR support OR practice OR practices OR service OR services))

**AND**

TI (Implement* OR disseminat* OR diffusion OR translation*) OR AB (Implement* OR disseminat* OR diffusion OR translation*)

**AND**

MH “Evidence-Based Practice+” OR TI (“evidence based” OR “empirically supported” OR “best practice” OR “best practices”) OR AB (“evidence based” OR “empirically supported” OR “best practice” OR “best practices”)

**AND**

LA English

**PscyInfo – 1,189 references**

DE "Stakeholder" OR DE "Client Participation" OR DE "Community Advocacy" OR DE "Government Programs" OR DE "Medicaid" OR DE "Medicare" OR DE "Social Security" OR DE "Welfare Services (Government)" OR DE "Caregivers" OR DE "Employer Attitudes" OR TI (Stakeholder OR stakeholders OR “key informant” OR “key informants” OR “engagement framework” OR “engagement method” OR “facilitating engagement” OR “community academic partnership” OR “community advocate” OR “community advocates”) OR AB (Stakeholder OR stakeholders OR “key informant” OR “key informants” OR “engagement framework” OR “engagement method” OR “facilitating engagement” OR “community academic partnership” OR “community advocate” OR “community advocates”) OR TI ((patient OR patients OR consumer OR consumers OR client OR clients OR public OR community OR communities OR customer OR customers OR agency OR agencies OR government* OR payer OR payers OR purchaser* OR insurance OR medicare OR Medicaid OR administrator* OR physician* OR clinician* OR provider* OR layperson* OR laypeople OR lay OR caregiver* OR employer*) N3 (engage* OR engaging OR collaborat* OR participat* OR partner* OR involve* OR input)) OR AB ((patient OR patients OR consumer OR consumers OR client OR clients OR public OR community OR communities OR customer OR customers OR agency OR agencies OR government* OR payer OR payers OR purchaser* OR insurance OR medicare OR Medicaid OR administrator* OR physician* OR clinician* OR provider* OR layperson* OR laypeople OR lay OR caregiver* OR employer*) N3 (engage* OR engaging OR collaborat* OR participat* OR partner* OR involve* OR input))

**AND**

DE "Mental Health Services" OR DE "Community Mental Health Services" OR DE "Preventive Mental Health Services" OR DE "Behavioral Health Services" OR DE "Psychotherapy" OR DE "Adlerian Psychotherapy" OR DE "Adolescent Psychotherapy" OR DE "Affirmative Therapy" OR DE "Analytical Psychotherapy" OR DE "Autogenic Training" OR DE "Brief Psychotherapy" OR DE "Brief Relational Therapy" OR DE "Child Psychotherapy" OR DE "Client Centered Therapy" OR DE "Conversion Therapy" OR DE "Couples Therapy" OR DE "Eclectic Psychotherapy" OR DE "Emotion Focused Therapy" OR DE "Existential Therapy" OR DE "Experiential Psychotherapy" OR DE "Expressive Psychotherapy" OR DE "Eye Movement Desensitization Therapy" OR DE "Feminist Therapy" OR DE "Geriatric Psychotherapy" OR DE "Gestalt Therapy" OR DE "Group Psychotherapy" OR DE "Guided Imagery" OR DE "Humanistic Psychotherapy" OR DE "Hypnotherapy" OR DE "Individual Psychotherapy" OR DE "Insight Therapy" OR DE "Integrative Psychotherapy" OR DE "Interpersonal Psychotherapy" OR DE "Logotherapy" OR DE "Narrative Therapy" OR DE "Network Therapy" OR DE "Persuasion Therapy" OR DE "Primal Therapy" OR DE "Psychoanalysis" OR DE "Psychodrama" OR DE "Psychodynamic Psychotherapy" OR DE "Psychotherapeutic Counseling" OR DE "Psychotherapeutic Techniques" OR DE "Rational Emotive Behavior Therapy" OR DE "Reality Therapy" OR DE "Relationship Therapy" OR DE "Solution Focused Therapy" OR DE "Strategic Therapy" OR DE "Supportive Psychotherapy" OR DE "Transactional Analysis" OR DE "Psychotherapeutic Techniques" OR DE "Active Listening" OR DE "Animal Assisted Therapy" OR DE "Autogenic Training" OR DE "Brief Relational Therapy" OR DE "Centering" OR DE "Cotherapy" OR DE "Dream Analysis" OR DE "Empty Chair Technique" OR DE "Ericksonian Psychotherapy" OR DE "Free Association" OR DE "Guided Imagery" OR DE "Life Review" OR DE "Mirroring" OR DE "Morita Therapy" OR DE "Motivational Interviewing" OR DE "Mutual Storytelling Technique" OR DE "Network Therapy" OR DE "Paradoxical Techniques" OR DE "Psychodrama" OR DE "Treatment" OR DE "Addiction Treatment" OR DE "Adjunctive Treatment" OR DE "Adventure Therapy" OR DE "Aftercare" OR DE "Alternative Medicine" OR DE "Anxiety Management" OR DE "Behavior Modification" OR DE "Bibliotherapy" OR DE "Caregiving" OR DE "Client Transfer" OR DE "Client Treatment Matching" OR DE "Cognitive Behavior Therapy" OR DE "Cognitive Stimulation Therapy" OR DE "Cognitive Techniques" OR DE "Computer Assisted Therapy" OR DE "Counseling" OR DE "Creative Arts Therapy" OR DE "Cross Cultural Treatment" OR DE "Disease Management" OR DE "Habilitation" OR DE "Health Care Services" OR DE "Horticulture Therapy" OR DE "Hospice" OR DE "Human Potential Movement" OR DE "Human Services" OR DE "Hydrotherapy" OR DE "Institutionalization" OR DE "Integrated Services" OR DE "Interdisciplinary Treatment Approach" OR DE "Intervention" OR DE "Involuntary Treatment" OR DE "Language Therapy" OR DE "Life Sustaining Treatment" OR DE "Maintenance Therapy" OR DE "Medical Treatment (General)" OR DE "Mental Health Programs" OR DE "Milieu Therapy" OR DE "Mind Body Therapy" OR DE "Mindfulness-Based Interventions" OR DE "Movement Therapy" OR DE "Multimodal Treatment Approach" OR DE "Multisystemic Therapy" OR DE "Outpatient Treatment" OR DE "Pain Management" OR DE "Partial Hospitalization" OR DE "Personal Therapy" OR DE "Physical Treatment Methods" OR DE "Private Practice" OR DE "Psychoeducation" OR DE "Psychotherapy" OR DE "Rehabilitation" OR DE "Relaxation Therapy" OR DE "Respite Care" OR DE "Self-Help Techniques" OR DE "Sex Therapy" OR DE "Social Casework" OR DE "Sociotherapy" OR DE "Speech Therapy" OR DE "Spiritual Care" OR DE "Symptoms Based Treatment" OR DE "Therapeutic Processes" OR DE "Trauma-Informed Care" OR DE "Trauma Treatment" OR DE "Treatment Guidelines" OR DE "Treatment Outcomes" OR DE "Treatment Planning" OR DE "Video-Based Interventions" OR TI (psychotherapy OR “behavioral therapy” OR “behavior therapy” OR “behaviour therapy” OR “behavioural therapy” OR “mental healthcare” OR “behavioral healthcare” OR “behavioural healthcare” OR (psychologic* OR psychiatric OR psychosocial OR behavioral OR behavioural OR “mental health” OR addiction) N5 (intervention OR treatment OR treatments OR therapy OR therapies OR care OR healthcare OR support OR practice OR practices OR service OR services)) OR AB (psychotherapy OR “behavioral therapy” OR “behavior therapy” OR “behaviour therapy” OR “behavioural therapy” OR “mental healthcare” OR “behavioral healthcare” OR “behavioural healthcare” OR (psychologic* OR psychiatric OR psychosocial OR behavioral OR behavioural OR “mental health” OR addiction) N5 (intervention OR treatment OR treatments OR therapy OR therapies OR care OR healthcare OR support OR practice OR practices OR service OR services))

**AND**

DE "Program Development" OR TI (Implement* OR disseminat* OR diffusion OR translation*) OR AB (Implement* OR disseminat* OR diffusion OR translation*)

**AND**

DE "Best Practices" OR DE "Evidence Based Practice" OR TI (“evidence based” OR “empirically supported” OR “best practice” OR “best practices”) OR AB (“evidence based” OR “empirically supported” OR “best practice” OR “best practices”)

AND

LA English

AND

PT Journal

**Embase – 1,317 references**

('community participation'/exp OR 'patient participation'/exp OR 'stakeholder engagement'/exp OR 'caregiver'/exp OR 'advocacy group'/exp OR (Stakeholder OR stakeholders OR “key informant” OR “key informants” OR “engagement framework” OR “engagement method” OR “facilitating engagement” OR “community academic partnership” OR “community advocate” OR “community advocates”):ti,ab OR ((patient OR patients OR consumer OR consumers OR client OR clients OR public OR community OR communities OR customer OR customers OR agency OR agencies OR government* OR payer OR payers OR purchaser* OR insurance OR medicare OR Medicaid OR administrator* OR physician* OR clinician* OR provider* OR layperson* OR laypeople OR lay OR caregiver* OR employer*) NEAR/3 (engage* OR engaging OR collaborat* OR participat* OR partner* OR involve* OR input)):ti,ab)

**AND**

('mental health care'/exp OR 'psychotherapy'/exp OR (psychotherapy OR “behavioral therapy” OR “behavior therapy” OR “behaviour therapy” OR “behavioural therapy” OR “mental healthcare” OR “behavioral healthcare” OR “behavioural healthcare”):ti,ab OR ((psychologic* OR psychiatric OR psychosocial OR behavioral OR behavioural OR “mental health” OR addiction) NEAR/5 (intervention OR treatment OR treatments OR therapy OR therapies OR care OR healthcare OR support OR practice OR practices OR service OR services)):ti,ab)

**AND**

('dissemination'/exp OR 'implementation'/exp OR 'implementation science'/exp OR 'translational research'/exp OR (Implement* OR disseminat* OR diffusion OR translation*):ti,ab)

**AND**

('evidence based practice'/exp OR (“evidence based” OR “empirically supported” OR “best practice” OR “best practices”):ti,ab)

AND

[English]/lim

NOT

‘conference abstract’/it

**CINAHL Complete (Ebsco) – 765 references**

MH "Community-Institutional Relations" OR MH "Stakeholder Participation" OR MH "Consumer Participation" OR MH "Caregivers" OR MH "Patient Advocacy" OR (MH "Insurance Carriers" OR TI (Stakeholder OR stakeholders OR “key informant” OR “key informants” OR “engagement framework” OR “engagement method” OR “facilitating engagement” OR “community academic partnership” OR “community advocate” OR “community advocates”) OR AB (Stakeholder OR stakeholders OR “key informant” OR “key informants” OR “engagement framework” OR “engagement method” OR “facilitating engagement” OR “community academic partnership” OR “community advocate” OR “community advocates”) OR TI ((patient OR patients OR consumer OR consumers OR client OR clients OR public OR community OR communities OR customer OR customers OR agency OR agencies OR government* OR payer OR payers OR purchaser* OR insurance OR medicare OR Medicaid OR administrator* OR physician* OR clinician* OR provider* OR layperson* OR laypeople OR lay OR caregiver* OR employer*) N3 (engage* OR engaging OR collaborat* OR participat* OR partner* OR involve* OR input)) OR AB ((patient OR patients OR consumer OR consumers OR client OR clients OR public OR community OR communities OR customer OR customers OR agency OR agencies OR government* OR payer OR payers OR purchaser* OR insurance OR medicare OR Medicaid OR administrator* OR physician* OR clinician* OR provider* OR layperson* OR laypeople OR lay OR caregiver* OR employer*) N3 (engage* OR engaging OR collaborat* OR participat* OR partner* OR involve* OR input))

**AND**

MH "Mental Health Services+" OR MH "Psychotherapy+" OR MH "Somatic Therapies, Psychiatric+" OR MH "Psychological Techniques+" OR TI (psychotherapy OR “behavioral therapy” OR “behavior therapy” OR “behaviour therapy” OR “behavioural therapy” OR “mental healthcare” OR “behavioral healthcare” OR “behavioural healthcare” OR (psychologic* OR psychiatric OR psychosocial OR behavioral OR behavioural OR “mental health” OR addiction) N5 (intervention OR treatment OR treatments OR therapy OR therapies OR care OR healthcare OR support OR practice OR practices OR service OR services)) OR AB (psychotherapy OR “behavioral therapy” OR “behavior therapy” OR “behaviour therapy” OR “behavioural therapy” OR “mental healthcare” OR “behavioral healthcare” OR “behavioural healthcare” OR (psychologic* OR psychiatric OR psychosocial OR behavioral OR behavioural OR “mental health” OR addiction) N5 (intervention OR treatment OR treatments OR therapy OR therapies OR care OR healthcare OR support OR practice OR practices OR service OR services))

**AND**

MH "Implementation Science" OR MH "Program Implementation" OR MH "Diffusion of Innovation+" OR TI (Implement* OR disseminat* OR diffusion OR translation*) OR AB (Implement* OR disseminat* OR diffusion OR translation*)

**AND**

MH "Professional Practice, Evidence-Based+" OR TI (“evidence based” OR “empirically supported” OR “best practice” OR “best practices”) OR AB (“evidence based” OR “empirically supported” OR “best practice” OR “best practices”)

AND

LA English

**ERIC (Ebsco) – 62 references**

DE "Stakeholders" OR DE "Learner Engagement" OR DE "Citizen Participation" OR DE "Teacher Collaboration" OR DE "Cooperative Planning" OR DE "Agency Cooperation" OR DE "School Community Relationship" OR TI (Stakeholder OR stakeholders OR “key informant” OR “key informants” OR “engagement framework” OR “engagement method” OR “facilitating engagement” OR “community academic partnership” OR “community advocate” OR “community advocates”) OR AB (Stakeholder OR stakeholders OR “key informant” OR “key informants” OR “engagement framework” OR “engagement method” OR “facilitating engagement” OR “community academic partnership” OR “community advocate” OR “community advocates”) OR TI ((patient OR patients OR consumer OR consumers OR client OR clients OR public OR community OR communities OR customer OR customers OR agency OR agencies OR government* OR payer OR payers OR purchaser* OR insurance OR medicare OR Medicaid OR administrator* OR physician* OR clinician* OR provider* OR layperson* OR laypeople OR lay OR caregiver* OR employer*) N3 (engage* OR engaging OR collaborat* OR participat* OR partner* OR involve* OR input)) OR AB ((patient OR patients OR consumer OR consumers OR client OR clients OR public OR community OR communities OR customer OR customers OR agency OR agencies OR government* OR payer OR payers OR purchaser* OR insurance OR medicare OR Medicaid OR administrator* OR physician* OR clinician* OR provider* OR layperson* OR laypeople OR lay OR caregiver* OR employer*) N3 (engage* OR engaging OR collaborat* OR participat* OR partner* OR involve* OR input))

**AND**

DE "Psychoeducational Methods" OR DE "Psychotherapy" OR DE "Milieu Therapy" OR DE "Relaxation Training" OR DE "Mental Health Programs" OR DE "Art Therapy" OR DE "Bibliotherapy" OR DE "Group Therapy" OR DE "Psychological Services" OR DE "Cognitive Restructuring" OR DE "Behavior Modification" OR DE "Contingency Management" OR DE "Desensitization" OR DE "Positive Behavior Supports" OR TI (psychotherapy OR “behavioral therapy” OR “behavior therapy” OR “behaviour therapy” OR “behavioural therapy” OR “mental healthcare” OR “behavioral healthcare” OR “behavioural healthcare” OR (psychologic* OR psychiatric OR psychosocial OR behavioral OR behavioural OR “mental health” OR addiction) N5 (intervention OR treatment OR treatments OR therapy OR therapies OR care OR healthcare OR support OR practice OR practices OR service OR services)) OR AB (psychotherapy OR “behavioral therapy” OR “behavior therapy” OR “behaviour therapy” OR “behavioural therapy” OR “mental healthcare” OR “behavioral healthcare” OR “behavioural healthcare” OR (psychologic* OR psychiatric OR psychosocial OR behavioral OR behavioural OR “mental health” OR addiction) N5 (intervention OR treatment OR treatments OR therapy OR therapies OR care OR healthcare OR support OR practice OR practices OR service OR services))

**AND**

DE "Program Implementation" OR TI (Implement* OR disseminat* OR diffusion OR translation*) OR AB (Implement* OR disseminat* OR diffusion OR translation*)

**AND**

DE "Evidence Based Practice" OR DE "Best Practices" OR TI (“evidence based” OR “empirically supported” OR “best practice” OR “best practices”) OR AB (“evidence based” OR “empirically supported” OR “best practice” OR “best practices”)

AND

LA English

AND

PU “Journal articles”

**Scopus – 1079 references**

TITLE-ABS-KEY-AUTH(Stakeholder OR stakeholders OR “key informant” OR “key informants” OR “engagement framework” OR “engagement method” OR “facilitating engagement” OR “community academic partnership” OR “community advocate” OR “community advocates” OR ((patient OR patients OR consumer OR consumers OR client OR clients OR public OR community OR communities OR customer OR customers OR agency OR agencies OR government* OR payer OR payers OR purchaser* OR insurance OR medicare OR Medicaid OR administrator* OR physician* OR clinician* OR provider* OR layperson* OR laypeople OR lay OR caregiver* OR employer*) W/3 (engage* OR engaging OR collaborat* OR participat* OR partner* OR involve* OR input)))

**AND**

TITLE-ABS-KEY-AUTH(psychotherapy OR “behavioral therapy” OR “behavior therapy” OR “behaviour therapy” OR “behavioural therapy” OR “mental healthcare” OR “behavioral healthcare” OR “behavioural healthcare” OR ((psychologic* OR psychiatric OR psychosocial OR behavioral OR behavioural OR “mental health” OR addiction) W/5 (intervention OR treatment OR treatments OR therapy OR therapies OR care OR healthcare OR support OR practice OR practices OR service OR services)))

**AND**

TITLE-ABS-KEY-AUTH(Implement* OR disseminat* OR diffusion OR translation*)

**AND**

TITLE-ABS-KEY-AUTH(“evidence based” OR “empirically supported” OR “best practice” OR “best practices”)

AND

LANGUAGE(English)

AND

DOCTYPE(ar OR cp OR ed OR le OR no OR re OR sh)

**Web of Science Core Collection (Clarivate) – 781 references**

TOPIC: Stakeholder OR stakeholders OR “key informant” OR “key informants” OR “engagement framework” OR “engagement method” OR “facilitating engagement” OR “community academic partnership” OR “community advocate” OR “community advocates” OR ((patient OR patients OR consumer OR consumers OR client OR clients OR public OR community OR communities OR customer OR customers OR agency OR agencies OR government* OR payer OR payers OR purchaser* OR insurance OR medicare OR Medicaid OR administrator* OR physician* OR clinician* OR provider* OR layperson* OR laypeople OR lay OR caregiver* OR employer*) NEAR/3 (engage* OR engaging OR collaborat* OR participat* OR partner* OR involve* OR input))

**AND**

TOPIC: psychotherapy OR “behavioral therapy” OR “behavior therapy” OR “behaviour therapy” OR “behavioural therapy” OR “mental healthcare” OR “behavioral healthcare” OR “behavioural healthcare” OR ((psychologic* OR psychiatric OR psychosocial OR behavioral OR behavioural OR “mental health” OR addiction) NEAR/5 (intervention OR treatment OR treatments OR therapy OR therapies OR care OR healthcare OR support OR practice OR practices OR service OR services))

**AND**

TOPIC: Implement* OR disseminat* OR diffusion OR translation*

**AND**

TOPIC: “evidence based” OR “empirically supported” OR “best practice” OR “best practices”

AND

LANGUAGE: English

AND

DOCUMENT TYPE: article, early access, editorial material, letter, proceedings paper, retraction, review
